# Case of Trismus

**Published:** 1847-06

**Authors:** 


*Case of Trismus.*—Mr. H. Smith reports, in the *Medical Times,* the following case, which came under his care, as house surgeon to King's College Hospital:—Elizabeth Croose, aged 25, unmarried, came to the hospital on the 3d of July, with the following symptoms: her teeth were completely closed; she was unable to separate them, by any effort on her part; there was great pain below the left ear, and underneath the left side of the lower jaw, which was greatly increased by any attempt to separate the teeth; she complained, also, of severe shooting pains on the left side of the face, and temporal region of the same side; the superficial glands under the jaw were enlarged and painful; her face was pale and bloodless, and presented the appearance of anxiety and bad health. I learnt from her the following history: twelve months ago, she had an attack of severe pain in the same situation, which was followed by almost perfect closing of the teeth; she had previously been in bad health; her business was that of a dressmaker, and she was confined much in-doors, at work; she applied to a medical man, who ordered fomentations to the face, and hot air baths: under which treatment she gradually improved, and she was able to open her teeth, but not to any great extent. She continued in rather delicate health until last January, when she had a recurrence of the attack; the teeth being very nearly closed, and the pain great. Her health became deranged; she suffered from languor and weakness; she was seen by a hospital surgeon, who ordered her tonics, viz. the cold bath, exercise and steel. Her health improved, gradual relaxation of the jaw took place, and she was enabled to separate her teeth well; she continued in pretty good health up to the present time, July 3d, when I saw her, laboring under the before-mentioned symptoms. Judging from the history of the case that the trismus was owing to some local irritation, acting upon a nervous and weak constitution, I determined to use local treatment, combined with general antispasmodics and stimulants: I ordered six leeches to be applied below the ear, to be followed by fomentations, and a mixture composed of Dec. Aloes. Co., Tinct. Asafœtidæ, and Spirit of Ammonia, and a sedative of Opium and Camphor at bed time.

July 4th. Has passed a more comfortable night from the opium; but she complains of great pain about the jaw, and the trismus is quite as obstinate; she can take nothing but liquids; the pulse is rapid and feeble, countenance very anxious, bowels confined, there appears to be no spasm of the masseter muscle: ordered a blister below the ear, and the raw surface afterwards to be sprinkled over with gr. ij. of muriate of morphia; wine and arrowroot, to be taken occasionally. Mag. Sulph., one oz., to be added to the mixture.

July 5th. Has passed a very restless night; is in very great pain; she has only experienced temporary relief from the local applications; the pain attacks her in paroxysms, and her sufferings at those periods appear dreadful; she is considerably exhausted, and in an anxious state of mind, the teeth keep firmly closed together. She complains of soreness in the throat, and experiences difficulty in swallowing liquid; the pulse is rapid and feeble; but there is no febrile disturbance. The bowels have been well opened.

I determined to try the effect of cold water, poured from a height upon her face, but it did not appear to produce any effect. As she was much exhausted, and could take no solid nourishment, I ordered her to have wine and arrowroot frequently.

July 6th. On entering the room, to my great surprise, I found her sitting up with a cheerful countenance. She expressed herself much better, and I was at a loss to account for her sudden improvement, when she gave me the following account: Suddenly, in the night, she *felt something burst* in her mouth, and found herself almost choked by a quantity of matter, which she was unable to eject; this was followed by great relief to the pain, and relaxation of the jaw. She was enabled to eat some solid food. The teeth have separated, but not to any extent; she still complains of great languor, and she suffers pain under the jaw still, but it is not severe. The superficial glands are enlarged and tender. I ordered four leeches to be applied, and a dose of quinine to be taken twice daily, and what nourishment she was able.

July 8th. Is much better, she can open her teeth still more, she has only some occasional shooting pains on the left side of the face; empl. hyttæ below the ear; she eats solid food, and is getting stronger.

July 12th. Is rapidly improving; she has been able to go out for a walk; her strength is returning; the teeth have separated more, and she does not complain of pain.

July 27th. I happened to see this young woman, when I found her health restored; her teeth have separated further, but not to the extent that they had before her last attack.

This case presents some interesting features. I believe it is a rare occurrence to meet with perfect trismus alone, except in hysteria. Contraction of the jaws occasionally results from inflammation of the joint, or from caries of the bone, but these cases are generally permanent.* From the sudden

* Cases precisely similar to the above are daily occurring to us as dental surgeons; the case, unquestionably, presents nothing more than the difficulty frequently experienced in the dens sapientiæ passing through the gum.—*Ed.*

termination of this case, from the symptoms of irritation about the jaw, and from the account she gave me, I think there can be no doubt that some abscess had formed about the jaw, and had suddenly burst. Although I suspected some mischief then, I must confess that I considered, from the previous history, and from her having been cured in the last attack solely by means calculated to improve her general health, that it chiefly depended on constitutional causes, therefore I chiefly attended to the constitutional treatment, although I did not neglect the local treatment, by leeches and blistering, which probably had the effect of bringing about a more speedy resolution of the abscess.

